# Social marketing interventions to promote physical activity among 60 years and older: a systematic review of the literature

**DOI:** 10.1186/s12889-020-09386-x

**Published:** 2020-08-28

**Authors:** Luc Goethals, Nathalie Barth, David Hupin, Michael S. Mulvey, Frederic Roche, Karine Gallopel-Morvan, Bienvenu Bongue

**Affiliations:** 1 Laboratoire SNA EPIS EA 4607, Université Jean Monnet, Saint-Étienne, France; 2 Gerontopole AURA, Saint-Etienne, France; 3 Service de physiologie, Clinique et de l’exercice, CHU de Saint-Etienne, Saint-Etienne, France; 4 Telfer School of Management, University of Ottawa, Ottawa, Ontario Canada; 5 Ecole des Hautes Etudes en Santé Publique / EA 7348 MOS, Rennes, France; 6 Centre Technique d’Appui et de Formation (CETAF), Saint-Étienne, France

**Keywords:** Social marketing, Physical activity, Older adults, Interventions, Systematic review

## Abstract

**Background:**

Falls are a significant source of morbidity in people aged 65 and over, affecting one in three people in this age group. The scientific evidence indicates that physical activity is the most effective method for preventing falls among seniors. Although public health professionals often use social marketing to design and plan successful interventions, its use to promote physical activity and prevent falls among older people remains low. This article aims to provide a new systematic literature review of social marketing interventions promoting physical activity and targeting people aged 60 and over.

**Methods:**

Following CRD’s guidance and PRISMA guidelines, we searched between January 2008 and July 2019 for relevant articles in five primary databases using predefined search and inclusion criteria. Two independent reviewers analysed the selected articles to identify evidence of the seven social marketing benchmark criteria, defined by experts in the field as the common elements that contribute to social marketing success.

**Results:**

The final review included nine studies. Of the studies selected, three specifically targeted over 60-year-olds, whereas the others segmented the population into several age-based subcategories, including over 60-year-olds. Eight studies highlighted positive results for the participants with an increase in participation or an increase in physical activity level. None of the nine studies selected for this systematic review implemented the entire social marketing approach.

**Conclusion:**

Few published interventions use the seven social marketing criteria. Further research is required to encourage uptake and inclusion in successful social marketing interventions to increase program effectiveness in this target population.

## Background

Population ageing is a global phenomenon and presents many public health challenges in particular through the development of systems for long-term care or the creation of environments adapted to the elderly [1]. Between 2015 and 2050, projections show that the proportion of over 60 years old in the world population should rise from 12 to 22%, representing nearly two billion people [1]. Life expectancy is increasing, but improving quality of life through preserving functional ability by practicing, for example, physical activity is important to permit older adults to maintain their level of independence [2, 3].

Falls are a significant source of morbidity in people aged 65 and over, affecting one in three people in this age group [4]. A recent systematic review and meta-analysis examined the association of physical activity and the risk of falling for older adults. It concluded that the risk of being a recurrent faller (two or more self-reported falls over the follow-up period of 12–36 months) was 39% higher in those older adults with the lowest levels of physical activity [5]. Each year in the USA, the cost of non-fatal fall injuries among adults age 65 and over is about $50 billion. The cost of fatal falls is about $754 million [6].

Physical activity (PA) represents the most effective method for preventing falls among older people [4, 7, 8], while PA interventions focusing on balance are the most effective way of avoiding loss of autonomy among older people living at home [4, 9]. There is evidence that these balance-based programs can be cost-effective [10]. These programs produce excellent results for the health of the older people and improve, in particular, the remaining life of the participants [4, 10, 11].

However, though there is now agreement that PA is useful in practice, there are many barriers to participation [12, 13]. Indeed, several such barriers exist among the elderly, such as a belief of no longer being able to participate because of a loss of physical capacity, an image of sports as being for young, healthy people, and poor awareness of the tailored activities on offer [12–14]. That is maybe why, all in all, only a small number of seniors participate in PA for fall prevention [15, 16].

Different methods are available for educators to increase PA for seniors. This paper focuses on social marketing. Social marketing is one of the most widely used methods for promoting behaviours that benefit the health of the population [12, 13]. Social marketing is *“the adaptation of commercial marketing technologies to programs designed to influence the voluntary behavior of target audiences to improve their personal welfare and that of the society”* [17]. Social marketing proposes to appropriate the principles and approach from commercial marketing, used in particular by private companies, to set up more effective prevention programs [18]. Seven criteria guide the social marketing method: setting the aim of the intervention, formative research (analysis of the target audience), of the “competition”, segmentation of the target audience, offering an “exchange”, setting the marketing mix (the 4Ps), and evaluation of the program [19–21].

Successful health interventions based on social marketing principles have been achieved in domains such as smoking prevention, obesity prevention, as well as in the reduction of the use of alcohol have [14–16]. Moreover, these social marketing initiatives have been effective in different age groups, including adolescents [22], adults [23], and older people [24].

Although this method has shown its value in other contexts or groups of age, its use in getting seniors to take part in regular PA remains limited [25]. Recent systematic reviews have focused on identifying social marketing interventions to increase physical activity level among older adults [25]. No available reviews have focused specifically on researching and selecting social marketing interventions to increase physical activity level among older adults that use a minimum of social marketing criteria to ensure the quality of the intervention. However, as a result of differences in reporting intervention outcomes, a meta-analysis could not be undertaken [24].

### Aim and review question

The aim of our systematic review was to provide an update systematic literature review of social marketing interventions to support PA among over 60-year-olds.

Our research question is: “Could social marketing interventions increase the participation level of older adults in PA program?”. The contribution of our systematic review is (i) to update a previous review [24] and (ii) to add another selection criterion by selecting only interventions that have used at least four of the seven social marketing benchmarks. We chose to select only those studies that used at least four social marketing criteria to ensure the quality of the approach they proposed.

## Method

### Design

A structured systematic literature search was performed in September, 2019, using established standards (CRD’s guidance and PRISMA guidelines [26–28] and integrate how the protocol and focus for the review developed in this. This review protocol aimed to limit bias and ensure the best objectivity of the systematic review (Additional file 1).

### Data collection

We analysed social marketing interventions that aimed to increase participation levels of people aged 60 years and over to PA program in French or English in peer-reviewed journals published between January 2008 and July 2019.

### Data sources and search strategies

We executed the search using five databases with extensive coverage of the public health literature: Web of Science, PubMed, EBSCOhost, ScienceDirect, and BASE. Table 1 reports the search strategies and keywords used for each of the databases along with the number of studies found.

**Table 1:** Search strategies and keywords used

Databases	Search strategy	Results
PubMed	((((physical+activit* OR exercis*))) AND ((intervention* OR Randomi#ed. Controlled Trial OR evaluation OR trial OR campaign* OR program* OR study OR studies))) AND social marketing	359
	Filters: Full text available; Publication date from 2008/01/01 to 2019/07/01; Humans; English; French	
Web Of Science	TS = (physical+activit* OR exercis*) AND TS = (intervention* OR Randomized Controlled Trial OR evaluation OR trial OR campaign* OR program* OR study OR studies) AND (TS = social marketing)	529
	Language**:** (English OR French) Timespan = 2008–2019; Refined by: Document types = Article	
EBSCOhost	(physical+activit* OR exercis*) AND (intervention* OR Randomized Controlled Trial OR evaluation OR trial OR campaign* OR program* OR study OR studies) AND social marketing	169
	Filters: Full text available; Publication date from 2008/01/01 to 2019/07/01; English; French	
ScienceDirect	(“physical activity” OR exercise*) AND (intervention* OR “Randomized Controlled Trial” OR evaluation OR trial OR campaign* OR program* OR study OR studies) AND “social marketing”	446
	Filters: Year(s): 2008–2019; Articles types: review articles, research articles, and case reports,	
Bilefeld Academic Search Engine (BASE)	(physical+activit* OR exercis*) AND (intervention* OR Randomized Controlled Trial OR evaluation OR trial OR campaign* OR program* OR study OR studies) AND social marketing	221
	Filters: Year(s): 2008–2019; Articles types: Journal / Newspaper; English; French	
Total		1724

### Inclusion and exclusion criteria

All potentially relevant articles and records were imported into Zotero 5.0.73 citation management software. Articles were included if they proposed and evaluated a social marketing intervention aiming to increase physical activity level among people aged 60 and over. The exclusion criteria were: 1) papers that did not use at least four social marketing benchmarks; 2) interventions aimed at children and adults under 60; 3) interventions that did not target PA; 4) articles published in languages other than English or French.

A coding framework using existing definitions of social marketing delineated social marketing domains. Analysis of the selected articles focused on identifying evidence of the seven social marketing benchmark criteria defined by researchers in the field [19, 20, 29, 30]. Specifically:

#### Behavioural objective

Behavioural change was the primary objective of the intervention (i.e.*,* increase PA);

#### Formative research

Qualitative or quantitative studies were conducted on the target audiences to understand their characteristics, habits, and needs better;

#### Segmentation

The target audience was segmented based on common characteristics (e.g., sex, social background) and the interventions tailored to specific segments;

#### Exchange

Consider what the audience values and the price they paid; attends to the perceived/actual benefits and perceived/actual costs of engaging in the focal behaviour, taking part in more PA;

#### Marketing mix

The intervention uses elements of the marketing mix (the 4Ps: product, price, place, promotion) to achieve the objectives of promoting PA. It employs tools such as influencers (e.g., work with partners on the ground like associations, companies), cost (offer financial support mechanisms to combat financial or psychological barriers to adopting the behaviour), ease of access (facilitate access to services or products that enable a change in the target behaviour), and communication (promote the behaviour through the use of communication tools) [18];

#### Competition

A competitor analysis was performed (and factored into the program) to identify any issues that could hinder the adoption of the proposed effort to increase PA, for example, the existence of other organisations or competing programs if we take the point of view of a provider of PA for older adults. If we take the point of view of older adults it can be for example television and sedentary.;

#### Evaluation

An assessment of the program.

**Fig. 1:**
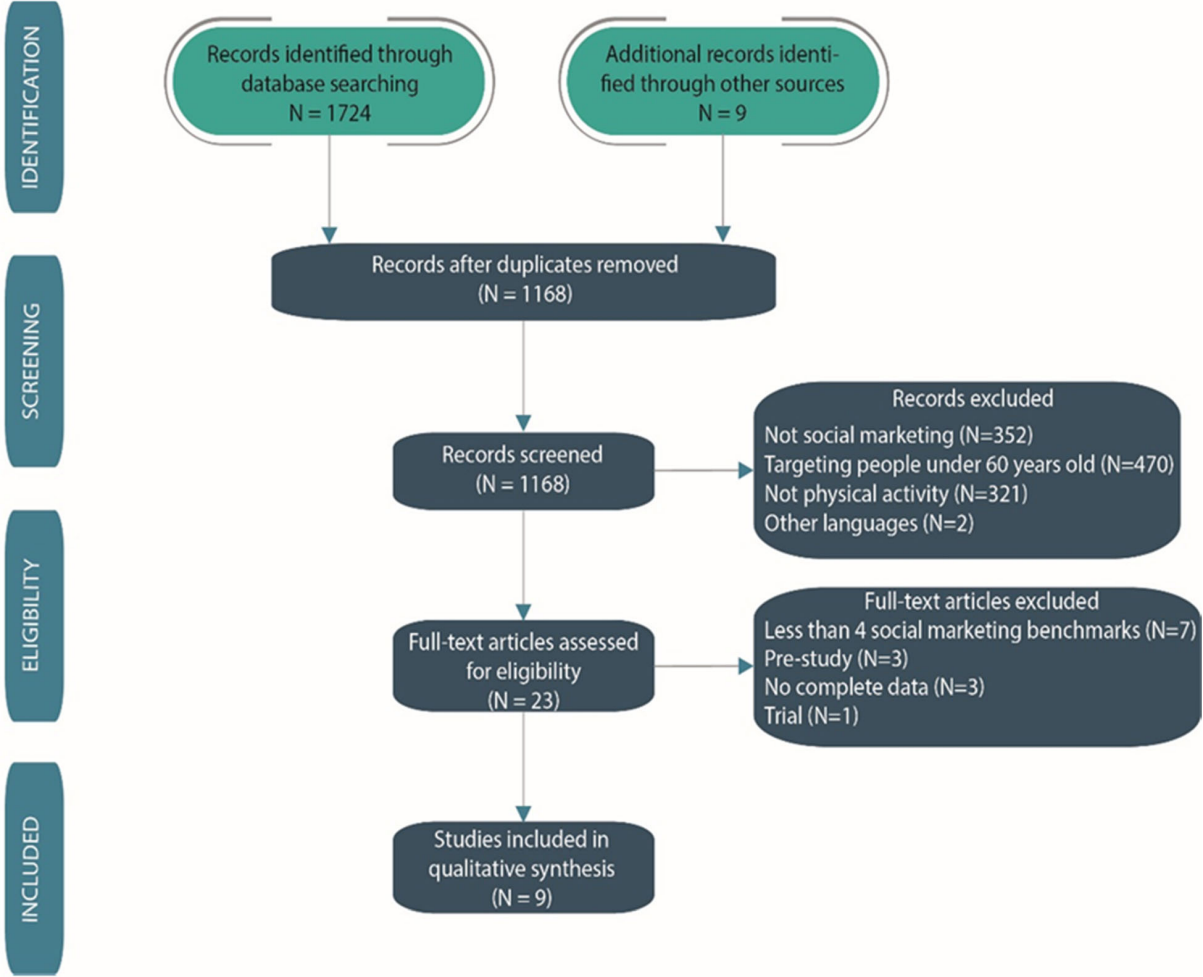
Flowchart of the literature review process

A total of 9 studies met the inclusion criteria (Fig. 1 summarises the literature review process, and the full list of studies can be found in Table 2).

**Table 2:** Studies included in the analysis

Reference	Intervention	Country	Duration of the social marketing campaign	Sample size	Interventions included	QAT score	Risk of bias
DiGuiseppi et al. (2014) [31]	N’Balance	USA	21 months	280 people	DiGuiseppi CG, Thoreson SR, Clark L, Goss CW, Marosits MJ, Currie DW, et al. Church-based social marketing to motivate older adults to take balance classes for fall prevention: Cluster randomized controlled trial. Prev Med. 2014;67:75–81.	80,9%	Low
Kamada et al. (2018) [32]	Communicate	Japan	5 years	4414 people	Kamada M, Kitayuguchi J, Abe T, Taguri M, Inoue S, Ishikawa Y, et al. Community-wide intervention and population-level physical activity: a 5-year cluster randomized trial. Int J Epidemiol. 012018;47 [2]:642–53.	78,6%	Low
Wilson et al. (2015) [33]	PATH	USA	2 years	434 people	Wilson DK, van Horn ML, Siceloff ER, Alia KA, St George SM, Lawman HG, et al. The Results of the « Positive Action for Today’s Health » (PATH) Trial for Increasing Walking and Physical Activity in Underserved African-American Communities. Annals of behavioral medicine: a publication of the Society of Behavioral Medicine. 2015;49 [3]:398–410.	71,4%	Medium
Withall et al. (2012) [34]	Fit and Fab	United-Kingdom	6 months	364 people	Withall J, Jago R, Fox KR. The effect a of community-based social marketing campaign on recruitment and retention of low-income groups into physical activity programmes - a controlled before-and-after study. BMC public health. 2012;12:836.	73,8%	Medium
Varma et al. (2015) [35]	Baltimore Experience Corps trial	USA	3 years	702 people	Varma VR, Tan EJ, Gross AL, Harris G, Romani W, Fried LP, et al. Effect of Community Volunteering on Physical Activity: A Randomized Controlled Trial. Am J Prev Med. Jan 2016;50 [1]:106–10.	66,7%	Low
Matsudo et al. (2002) [36]	The Agita São Paulo experience	Brazil	4 years	645 people	Matsudo V, Matsudo S, Andrade D, Araujo T, Andrade E, Oliveira LC de, et al. Promotion of physical activity in a developing country: The Agita São Paulo experience. Public Health Nutrition. 2002 Feb;5(1a):253–61.	57,1%	High
Reger-Nash et al. (2006) [37]	Wheeling,WV, BCWelch Walks	USA	2 months (four times)	3944 people	Reger-Nash B, Bauman A, Cooper L, Chey T, Simon KJ. Evaluating community wide walking interventions. Evaluation and Program Planning. 2006 Aug 1;29 [3]:251–9.	58,3%	Medium
Russell et al. (2007) [38]	Chef CharlesClub	USA	6 months	60 people	Russell C, Oakland MJ. Nutrition Education for Older Adults: The Chef Charles Club. Journal of Nutrition Education and Behavior. 2007 Jul 1;39 [4]:233–4.	52,4%	High
Richert et al. (2007) [39]	Move MoreDiabetes	USA	2 years	1500 people	Richert ML, Webb AJ, Morse NA, O’Toole ML, Brownson CA. Move More Diabetes. Diabetes Educ. 2007 Jun 1;33(S6):179S–184S.	71,4%	High

### Quality assessment and data extraction

Two independent reviewers analysed the selected articles to identify evidence of at least four of the seven social marketing benchmark criteria, defined by experts in the field as the common elements that contribute to social marketing success.

To take into account the general nature of studies and to avoid bias towards quantitative methods, we use a transparent and validated tool developed by Sirriyeh et al. [40]. The tool consists of 16 criteria each with a score between 0 and 3, with 3 being the best score.

For the description of the 16 criteria, see the supplementary material (Additional file 2). The fulfillment of each of the 16 criteria was assessed independently by two authors (and then consolidated by consensus) for each publication, based on the information provided in the evaluated document, and a score corresponding to the level of satisfactory fulfillment of the criteria, as described by Sirriyeh et al. [40], was given. For each item, the scores were added together and divided by the maximum possible score to reflect the paper’s overall quality score.

This is supplemented by an assessment of studies’ risk of bias in line with Cochrane guidelines [41] and PRISMA [27]. Criteria for risk of bias assessment included each social marketing benchmark. Each study received a summary of risk of bias score (high, medium, or low) based on Cochrane taxonomy [41]. The results can be found in Table 1.

Data extraction included the country of origin of the intervention, duration of the social marketing campaign, sample size, results of the intervention through adherence to the proposed physical activity programs, and increase in physical activity levels of participants.

A meta-analysis was not carried out given the nature of the study design and the differences in reporting intervention outcomes. A narrative synthesis of the results was carried out.

### Synthesis

Using existing definitions of social marketing, a coding framework was used that delineated the constituent domains of social marketing. Articles were organized according to each social marketing benchmark and we conducted a narrative synthesis of the selected studies.

## Results

Of the nine interventions, eight [31–38] increased participation in the activities offered or increased the level of PA. One study reported no effects [39].

Of the studies selected, three specifically targeted over 60-year-olds [31, 35, 38], whereas the others segmented the population into subcategories, one of which was over 60-year-olds.

Table 3 shows an assessment of the use of the seven social marketing benchmark criteria as defined by researchers in social marketing. None of the nine interventions selected for this literature review implemented the entire social marketing approach. In other words, none met all of the seven criteria presented above. Three of the nine interventions employed six of the criteria [31, 32, 36], five interventions used five [33–35, 37, 38], and one employed four [39].

**Table 3:** Assessing the use of the seven reference criteria of social marketing and the observed impact on the increase in physical activity

Interventions	Target	Behavioral change	Population study	Segmentation	Exchange	Marketing mix	Competition	Evaluation	Observed impact on the increase in physical activity
(DiGuiseppi et al., 2014) [31]	> 60 yrs	Yes	Yes	No	Yes	Yes	Yes	Yes	Yes
(Verma et al., 2016)	> 60 yrs	Yes	Yes	No	Yes	Yes	No	Yes	Yes
(Kamada et al., 2018) [32]	40–79 yrs	Yes	Yes	Yes	Yes	Yes	No	Yes	Yes
(Wilson et al., 2015) [33]	18–85 yrs	Yes	Yes	No	Yes	Yes	No	Yes	Yes
(Withall et al., 2012) [34]	≥18 yrs	Yes	Yes	No	Yes	Yes	No	Yes	Yes
(Matsudo et al., 2002) [36]	18 yrs. ≤ to > 60 yrs	Yes	Yes	Yes	No	Yes	Yes	Yes	Yes
(Russell and Oakland,.2007) [38]	> 60 yrs	Yes	Yes	No	No	Yes	Yes	Yes	Yes
(Reger-Nash et al., 2006) [37]	35–65 yrs	Yes	Yes	Yes	No	No	Yes	Yes	Yes
(Richert et al., 2007) [39]	30–70 yrs	Yes	Yes	No	No	Yes	No	Yes	No

yrs: years

### Behavioural objective

The nine interventions developed programs specified specific goals to change people’s actual behaviour. Specifically, Carolyn DiGuiseppi et al. [31] aimed to increase the attractiveness of balance classes (physical activity) for over 60-year-olds in selected churches. Dawn Wilson et al. [33] aimed to improve the walking level of low-income African-American communities in the study area. Janet Withall et al. [34] conducted a study that aimed to increase recruitment and adherence in a PA program in a low-income neighbourhood. Masamitsu Kamada et al. [32] aimed to increase the proportion of people aged 40 to 79 participating in aerobic, flexibility, and muscle-strengthening activities in the city of Unnan, Japan. Lastly, Vijay Varma et al. [35] aimed to increase walking levels among over 60-year-olds recruited as volunteers into public schools in the US city of Baltimore.

### Formative research

Formative research is crucial in a social marketing intervention [21] because it allows the social marketer to understand the target audience and the targeted behaviour better. All interventions conducted interview-based market studies to identify the barriers and facilitators of the target behaviour. Withall et al. [34] employed a mixed-method, using a questionnaire to assess people’s motivation to take part in group PA along with group interviews.

### Segmentation

Of the nine interventions, three reported the use of segmentation. In the study by Kamada et al. [32], the researchers used a model to determine a primary target segment for communications: women aged 60 to 79 years. They chose to emphasise three points: the total number of persons in the segment, the risk status, and the persuasibility of the segment. Reger-Nash et al. [37] created advertisements for the African American community by featuring African American actors to appeal to the regional minority population [25].

### Exchange

Only five interventions implemented the exchange concept by using incentives. In the study by DiGuiseppi et al. [31], participants received $5 if they took part in the classes on offer. The church leaders facilitated the transmission of messages and communications to the study’s target audience. Withall et al. [34] offered the first six weeks of sessions free of charge in their intervention, after which the price rose to £1 per session. A low price was used to attract low-income individuals to the meetings. In the study by Wilson et al. (2015), participants received a $20 gift card for each assessment period. Lastly, in the study by Varma et al. [35], participants received financial compensation for the time they volunteered as well as $25 for taking part in an assessment and $10 for a telephone interview. Also, the program permitted to offer social ties between participants in the programs.

### Marketing mix

Eight interventions used the marketing mix principles to set up their program. For example, in the intervention by DiGuiseppi et al. [31], the “product” was a fall prevention class, while the communication involved distributing flyers or newsletters promoting these classes. They selected churches as their location to facilitate access to the target audience, and the cost of the classes was $20. Price is one of the psychological barriers to taking part in PA. However, perceived costs include more than just the admission fee and also involve the number of sessions, their frequency, and the distance from home, not to mention the fear of falling or aggravating existing pain. Mindful of this, DiGuiseppi et al. reduced these barriers, offering classes in a safe, comfortable environment, and providing a schedule that suited the participants’ availability and preferences.

### Competition

Four interventions identified a facility that competed with their program. For DiGuiseppi et al. [31], that facility was a wellness centre offering exercise programs for over 60-year-olds.

### Evaluation

Of the interventions that had a positive impact on changing PA habits, that of Wilson et al. [33] assessed participation data and psychosocial data from participants at baseline as well as at 12, 18, and 24 months after the intervention. They revealed that uptake was higher among PATH trial participants who received communication on the PA program. Walking attendance was higher among these participants than among those who received no communication. Over nine months, the number of walkers rose from 40 to 400 per month in the social marketing intervention group [33].

Withall et al. [34] showed that enrollment in the different PA classes (dance, gym, and balance sessions) had increased since the start of the program, that attendance levels were steady, and that adherence was good.

Varma et al. [35] reported an increase in walking among women in the social marketing intervention group, with their daily step counts rising by a mean of 1500.

Kamada et al. [32] showed that their intervention increased the level of each type of PA promoted in their target population. That was a 5-year study, with the first positive results having appeared progressively over several years.

DiGuiseppi et al. [31] aimed to assess class participation and information retention by participants. Their intervention showed that the program implemented in the churches succeeded in motivating seniors to join PA classes. The participants were also more likely to remember information about fall prevention.

## Discussion

This article provided a systematic literature review of social marketing interventions to promote PA among over 60-year-olds. We found very few published studies on this topic; only nine interventions emerged from our search of five scientific databases. Of the nine interventions, eight concluded that social marketing was effective for increasing PA among the elderly.

The eight studies that reported a positive assessment of their programs believed their success to be mainly due to three factors. First, the use of social marketing techniques through the implementation of a number of criteria. A better understanding of core marketing concepts supports better planning, implementation, and effectiveness. Second, the funding of the activities, which made it possible to tackle financial barriers. Third, the building of social ties between participants in the programs, which provided them with support and interaction. For instance, Varma et al. [35] suggest that walking levels increased among women because they were taking part in a new voluntary activity.

Beyond effective social marketing programs, one failed. Kamada et al. (2013, 2015) state that their program did not succeed in demonstrating an increase in PA levels at 1 and 3 years because it was not comprehensive enough. For that reason, they suggested that a strategy of modifying the environment by improving the public transport system and building facilities specifically for PA could facilitate the meeting of recommended PA levels. Five years after the launch of the study, Kamada et al. [32] showed that their intervention had had a positive effect.

Eight interventions identified in our systematic review used the marketing mix and its “4Ps”: Price, Place, Promotion, and Product. The 4Ps represent a group of operational areas for developing strategies and tactics. This theory has been consensually accepted. However, Gallopel-Morvan et al. [20] advance five criteria they deem more suited to social marketing and behavioural change: Influencers, Behaviour, Cost, Ease of Access, and Communication. Notably, social marketers have a range of marketing tools at their disposal and use them in interventions.

The segmentation variables reported in the PA studies focused on basic descriptors of demographics (who?) and geography (where?). Although tried and tested, a narrow focus on markers as sex and gender can limit the kinds of insight required to develop an effective marketing strategy [42]. For instance, psychographic segmentation addresses the motivational concerns of target consumers and is widely used to develop user personas and profiles. Research on consumer personality traits, values, attitudes, interests, and lifestyles can identify what motivates targeted behaviours. Such insights have informed interventions that target segments of young adults who use tobacco [43, 44], for example Lisha et al.’s [44] study that identified distinct tobacco use risk patterns across different groups of young adults (Hipster, Country, Hip Hop, Partier, Homebody, and Young Professional). Research on the mindsets of older people and their views on physical activity are both lacking and needed.

Furthermore, implementing segmentation schemes that place a greater emphasis on actual consumer behaviour holds great potential [21]. Marketing involves exchanges, and consumers make decisions not only about what they “get” (e.g., products or services) but also about what they “give up” (e.g., allocation of time, effort, money, comfort, autonomy, privacy, social reputation). Social marketers ought to pay close attention to the nuances of behaviour and heed segment-level differences in benefits sought and willingness to pay [45], purchase and use occasions [46] or even patterns of social media sharing [47]. Used together, psychographic and behavioural segmentation can help to better understand and target messages for high-risk subgroups [44] and inform the development of targeted messages that echo a target audience’s existing views and practices, and therefore, produce more powerful persuasive effects [48].

Future research might consider looking at the dynamics of influence within the social networks of older people, given that exercise is often initiated and enacted with other people. A networked view of consumption can yield innovative intervention strategies. For example, consider the people implicated in mothers’ breastfeeding decisions [49]. Breastfeeding initiation and cessation decisions are shaped by varied aspects of the mother-infant relationship, guided by different healthcare professionals, muddled by mother-other child duties, enabled by spousal or caregiver support, and impacted by the (re) actions of other people in the workplace and public sphere. In a similar vein, research has leveraged insights about peer crowd affiliation to promote smoking cessation [50]. A consumption ensemble approach [51] could inform and inspire the development of sociologically-based intervention strategies.

The measurement of “daily step counts” by Varma *and al* [35]. is an excellent example of how technology (wearable devices) can generate performance feedback to both motivate participants and to assess the success of interventions. Applications of wearable technologies in healthcare had recently gained considerable interest from researchers and industry alike. Similarly, the retailing and services marketing literature increasingly look to self-service technologies as ways to improve service experiences while achieving operational efficiencies.

It is difficult to say whether social marketing is useful in promoting PA among seniors since none of the nine interventions selected used the entire approach (i.e., all seven benchmark criteria). Haruka Fujihira et al. [25] showed that the more a program uses the social marketing benchmark criteria defined by A.R. Andreasen, the more it becomes effective at changing the behavioural habits of target audiences. Literature reviews such as those by Julia Carins and Sharyn Rundle-Thiele [52], Bo Pang et al. [53], and Martine Stead et al. [54] have classified social marketing interventions according to the same benchmark criteria as Andreasen. They have reported similar findings to those of Fujihira (2015) [25].

Our literature review is based on the classic and widely-cited social marketing benchmark criteria defined by A.R. Andreasen [21]. Moreover, we added a seventh criterion, evaluation. Actually, other researchers, however, have expanded the theory. Observers have noted that many social marketing programs are poorly assessed or are not evaluated at all [55]. Accordingly, Gallopel-Morvan et al. [20] add a seventh criterion, evaluation, as a fundamental step in the process of a campaign using social marketing theory [20]. Among other things, program evaluation can provide valuable information about program design and implementation, and whether or not the program succeeded in changing the targeted behaviour [56].

Still, to improve the effectiveness of prevention campaigns using social marketing, other researchers adopt the eight criteria recommended by the UK National Social Marketing Centre [42, 43]. Among the additional criteria, one relates to the use of behavioural theory in social marketing intervention. Thus, recent work shows that when the theory of behaviour change is integrated into the construction of interventions, additional positive effects of the changes are noted [53, 57]. It is in this sense that the work of Sharyn Rundle-Thiele et al. [58] also goes, explaining that current research focuses on the explanation of individual behaviours and not on behaviour change. Moreover, the authors proposed ten social marketing theory development goals to “assist social marketers to develop new ways of thinking that will deliver the theory and evidence base needed to outline what practitioners and policymakers should do to effect change” [58]. The social marketing method is a young discipline. Today, several theories are in development. The most recent theories state that social marketing criteria should no longer be used as a simple tick-box checklist, but as a set of interrelated concepts [59].

Additionally, the papers that we selected in this review comprise certain limitations in terms of the assessment method, group heterogeneity, and contamination risk. The assessment models chosen preclude any definite direct attribution of the positive results to any one part of the intervention or any combination of activities. In some studies, discrepancies between the intervention and control groups interfered with the assessment of change between them. Withall et al. [34] suggested that the groups had differences (particularly in terms of age and ethnic origin), which prevented them from making direct comparisons during the assessment. Kamada et al. (2015) thought that the results of their study were contaminated because the control group may have been exposed to the social marketing campaign developed for the intervention group. This unintended exposure may have been due to the geographical proximity of the groups and word-of-mouth. Moreover, the criteria selected for assessing the prevention programs were not always the same, making it difficult to compare the interventions, or were insufficient to measure effectiveness. For instance, in the study by Withall et al. [34], the criteria only gauged PA program participation without measuring PA levels, PA intensity, or biological markers.

Some limitations of this study are to be highlighted. First, there is a publication bias because negative studies were probably less likely to be published. Second, our systematic literature review excludes public health and community-based interventions, which perhaps use social marketing techniques.

Different age groups (children, adults, seniors) have specific characteristics and guidelines in terms of public health and necessitate that separate social marketing interventions be designed for each one. Given both the lack of studies on using social marketing principles to promote PA in seniors, there is a need to develop more social marketing intervention promoting physical activity and targeting older people [25].

## Conclusion

Since PA has been identified as one of the effective interventions for healthy ageing [60] and since a large proportion of seniors are not active enough, targeted social marketing programs could help prevent numerous health problems associated with inactivity [25, 61]. As shown in this literature review, social marketing may have the potential to be useful for promoting PA among seniors. However, the studies we identified are not amenable to meta-analysis due to the heterogeneity of theoretical backgrounds, diverse treatment interventions, and reliance on different measures [62]. Further research and more studies are needed to advance our understanding of the vital link between physical activity and seniors’ health. By integrating social marketing criteria into intervention programs, we can better identify the drivers of intervention success and the impact of our efforts.

## Supplementary information

**Additional file 1.** Review protocol.

**Additional file 2.** Summary of methodological quality scores.

## Data Availability

The datasets analysed for the current study are available from the corresponding author on reasonable request.

## Abbreviations

CRD: Centre for Reviews and Dissemination
PA: Physical Activity
PRISMA: Preferred Reporting Items for Systematic Reviews and Meta-Analyses
UK: United Kingdom
USA: United States of America
