# Correction to: Social marketing interventions to promote physical activity among 60 years and older: a systematic review of the literature

**DOI:** 10.1186/s12889-020-09742-x

**Published:** 2020-10-28

**Authors:** Luc Goethals, Nathalie Barth, David Hupin, Michael S. Mulvey, Frederic Roche, Karine Gallopel-Morvan, Bienvenu Bongue

**Affiliations:** 1 Laboratoire SNA EPIS EA 4607, Université Jean Monnet, Saint-Étienne, France; 2 Service de Physiologie, Clinique et de l’exercice, CHU de Saint-Etienne, Saint-Etienne, France; 3 Telfer School of Management, University of Ottawa, Ottawa, Ontario Canada; 4 Ecole des Hautes Etudes en Santé Publique / EA 7348 MOS, Rennes, France; 5 Centre Technique d’Appui et de Formation (CETAF), Saint-Étienne, France

**Correction to: BMC Public Health 20, 1312 (2020)**

**https://doi.org/10.1186/s12889-020-09386-x**

It was highlighted that the original article [1] contained an error in Table 3. Also, two previous studies (Kamada et al. 2013, Kamada et al. 2015) were mentioned in the Discussion section but were missing from the reference list. This Correction article shows the correct Table 3 and the incorrect and correct citations.

**Table 3:** Assessing the use of the seven reference criteria of social marketing and the observed impact on the increase in physical activity

Interventions	Target	Behavioral change	Population study	Segmentation	Exchange	Marketing mix	Competition	Evaluation	Observed impact on the increase in physical activity
(DiGuiseppi et al. 2014)	> 60 yrs	Yes	Yes	No	Yes	Yes	Yes	Yes	Yes
(Verma et al. 2016)	> 60 yrs	Yes	Yes	No	Yes	Yes	No	Yes	Yes
(Kamada et al. 2018)	40–79 yrs	Yes	Yes	Yes	Yes	Yes	**Yes**	Yes	Yes
(Wilson et al. 2015)	18–85 yrs	Yes	Yes	No	Yes	Yes	No	Yes	Yes
(Withall et al. 2012)	≥18 yrs	Yes	Yes	No	Yes	Yes	No	Yes	Yes
(Matsudo et al. 2002)	18 yrs. ≤ to > 60 yrs	Yes	Yes	Yes	No	Yes	Yes	Yes	Yes
(Russell and Oakland 2007)	> 60 yrs	Yes	Yes	No	No	Yes	Yes	Yes	Yes
(Reger-Nash et al. 2006)	35–65 yrs	Yes	Yes	Yes	No	No	Yes	Yes	Yes
(Richert et al. 2007)	30–70 yrs	Yes	Yes	No	No	Yes	No	Yes	No

*yrs* years

**Incorrect:**

Abstract: “None of the nine studies selected for this systematic review implemented the entire social marketing approach.”

Results: Competition “Four interventions...”

Table 3: (Kamada et al. 2018: Competition: No)

Discussion: “It is difficult to say whether social marketing is useful in promoting PA among seniors since none of the nine interventions selected used the entire approach (i.e., all seven benchmark criteria).”

**Correct:**

Abstract: “Only one of the nine studies selected for this systematic review implemented the entire social marketing approach”

Results: **Competition**: Five interventions identified a facility that competed with their program.

Table 3: Kamada et al. 2018: Competition: Yes

Beyond effective social marketing programs, one failed. Kamada et al. [2, 3] state that their program did not succeed in demonstrating an increase in PA levels at 1 and 3 years because it was not comprehensive enough.

Discussion: “It is difficult to say whether social marketing is useful in promoting PA among seniors since only one of the nine interventions selected used the entire approach (i.e., all seven benchmark criteria).”
