# Supplementary material for: Social marketing interventions to promote physical activity among 60 years and older: a systematic review of the literature
Source: BMC Public Health. 2020 Aug 28;20:1312. doi: 10.1186/s12889-020-09386-x (PMC7456007; doi:10.1186/s12889-020-09386-x)
Supplement: Supplementary file 1 — Additional file 1. Review protocol. [file 12889_2020_9386_MOESM1_ESM.docx]

**Additional file 1 -** Review protocol

**Review question**

We aimed to provide a new systematic literature review of social marketing interventions to support PA among over 60-year-olds.

The review questions is:

- “Could social marketing interventions increase participation level of older adults in PA program?”

**Search**

We analysed social marketing interventions that aimed to increase PA among over 60-year-olds in French or English in peer-reviewed journals published between January 2008 and July 2019.

We executed the search using five databases with extensive coverage of the public health literature: Web of Science, PubMed, EBSCOhost, ScienceDirect, and BASE.

Search strategy for PubMed

((((physical+activit* OR exercis*))) AND ((intervention* OR Randomi#ed Controlled Trial OR evaluation OR trial OR campaign* OR program* OR study OR studies))) AND social marketing

Filters: Full text available; Publication date from 2008/01/01 to 2019/07/01; Humans; English; French

**Types of study to be included**

Articles were considered for inclusion if they proposed and evaluated a social marketing intervention aiming to increase physical activity level among people aged 60 and over. The search focused on articles written in English or French. The exclusion criteria were: 1) papers that did not use at least 4 social marketing benchmarks; 2) interventions aimed at children and adults under 60; 3) interventions that did not target PA; 4) articles published in languages other than English or French.

**Condition or domain being studied**

The review investigated the use of the social marketing method to promote physical activity among 60 years and older*.* The social marketing method has seven benchmarks (Behavioral objective, Formative research, Segmentation, Exchange, Marketing mix, Competition, Evaluation). We included articles that used at least four of the seven benchmarks.

**Participants/population**

The review included articles with people aged 60 and over. This threshold is in line with the World Health Organisation's (WHO) definition of older adults.

**Intervention(s), exposure(s)**

Not applicable.

**Comparator(s)/control**

Not applicable.

**Main outcome(s)**

Studies were required to include a description of the use of each social marketing benchmark.

**Data extraction (selection and coding)**

Full articles were assessed to ensure that studies met the inclusion/exclusion criteria for the type of study, construct being studied, participants, interventions and outcomes. For this reason, only studies for which the full-text version was available in English and French were included in this review. Two reviewers (LG and DH) conducted each phase of the selection process, e.g. eligibility and inclusion. The other members of the review team supervised the selection process.

Data were extracted from eligible studies using a uniform data extraction form. Data on social marketing benchmark was extracted, focusing on each benchmark.

**Risk of bias (quality) assessment**

Two independent coders analysed the selected articles to identify evidence of the seven social marketing benchmark criteria, defined by experts in the field as the common elements that contribute to social marketing success.

**Strategy for data synthesis**

Using existing definitions of social marketing, a coding framework was used that delineated the constituent domains of social marketing. Articles were organized according to each social marketing benchmark and we conduct a narrative synthesis of the selected studies.

**Analysis of subgroups or subsets**

There were not enough methodologically homogeneous studies to conduct meta-analyses.
