# Supplementary material for: Social marketing interventions to promote physical activity among 60 years and older: a systematic review of the literature
Source: BMC Public Health. 2020 Aug 28;20:1312. doi: 10.1186/s12889-020-09386-x (PMC7456007; doi:10.1186/s12889-020-09386-x)
Supplement: Supplementary file 2 — Additional file 2. Summary of methodological quality scores. [file 12889_2020_9386_MOESM2_ESM.docx]

**Additional file 2**: Summary of methodological quality scores

| Criteria | DiGuiseppi et *al*. (2014) | Kamada et a*l*. (2018) | Wilson et *al*. (2015) | Withall et *al*. (2012) | Varma et *al*. (2015) | Matsudo et *al*. (2002) | Reger-Nash et *al*. (2006) | Russell et *al*. (2007) | Richert et *al*. (2007) |
| --- | --- | --- | --- | --- | --- | --- | --- | --- | --- |
| Explicit theoretical framework | 3 | 2 | 2 | 2 | 1 | 3 | 2 | 1 | 3 |
| Statement of aims/objectives in main body of report | 3 | 3 | 3 | 3 | 3 | 3 | 3 | 3 | 3 |
| Clear description of research setting | 3 | 3 | 3 | 3 | 3 | 3 | 3 | 3 | 3 |
| Evidence of sample size considered in terms of analysis | 2 | 3 | 1 | 1 | 3 | 1 | 1 | 1 | 1 |
| Representative sample of target group of a reasonable size | 3 | 3 | 3 | 2 | 3 | 1 | 1 | 1 | 1 |
| Description of procedure for data collection | 3 | 3 | 3 | 3 | 3 | 3 | 3 | 3 | 3 |
| Rationale for choice of data collection tool(s) | 1 | 2 | 3 | 3 | 1 | 1 | 1 | 1 | 2 |
| Detailed recruitment data | 3 | 1 | 2 | 2 | 1 | 1 | 1 | 1 | 2 |
| Statistical assessment of reliability and validity of measurement tool(s)  (Quantitative only) | 1 | 1 | 2 | 2 | 1 | 1 | 1 | 1 | 2 |
| Fit between stated research question and method of data collection  (Quantitative only) | 3 | 3 | 2 | 3 | 3 | 2 | 2 | 2 | 3 |
| Fit between stated research question and format and content of data collection tool e.g. interview schedule  (Qualitative only) |  |  |  |  |  |  | 2 |  |  |
| Fit between research question and method of analysis  (Quantitative only) | 3 | 3 | 2 | 3 | 3 | 2 | 2 | 2 | 3 |
| Good justification for analytic method selected | 2 | 3 | 2 | 1 | 1 | 1 | 2 | 1 | 2 |
| Assessment of reliability of analytic process  (Qualitative only) |  |  |  |  |  |  | 2 |  |  |
| Evidence of user involvement in design | 2 | 1 | 1 | 1 | 1 | 1 | 1 | 1 | 1 |
| Strengths and limitations critically discussed | 2 | 2 | 1 | 2 | 1 | 1 | 1 | 1 | 1 |
| Score total/maximum score possible | 34/42 | 33/42 | 30/42 | 31/42 | 28/42 | 24/42 | 28/48 | 22/42 | 30/42 |
